# Author Correction: Layer 4 of mouse neocortex differs in cell types and circuit organization between sensory areas

**DOI:** 10.1038/s41467-019-12769-3

**Published:** 2019-11-04

**Authors:** Federico Scala, Dmitry Kobak, Shen Shan, Yves Bernaerts, Sophie Laturnus, Cathryn Rene Cadwell, Leonard Hartmanis, Emmanouil Froudarakis, Jesus Ramon Castro, Zheng Huan Tan, Stelios Papadopoulos, Saumil Surendra Patel, Rickard Sandberg, Philipp Berens, Xiaolong Jiang, Andreas Savas Tolias

**Affiliations:** 1 Center for Neuroscience and Artificial Intelligence, Baylor College of Medicine, Houston, TX USA; 2 Department of Neuroscience, Baylor College of Medicine, Houston, TX USA; 3 Institute for Ophthalmic Research, University of Tübingen, Tübingen, Germany; 4 Department of Anatomic Pathology, University of California San Francisco, San Francisco, CA USA; 5 Department of Cell and Molecular Biology, Karolinska Institutet, Stockholm, Sweden; 6 Department of Computer Science, University of Tübingen, Tübingen, Germany; 7 Jan and Dan Duncan Neurological Research Institute at Texas Children’s Hospital, Houston, TX USA; 8 Department of Electrical and Computational Engineering, Rice University, Houston, TX USA

**Keywords:** Neural circuits, Visual system

Correction to: *Nature Communications* 10.1038/s41467-019-12058-z, published online 13 September 2019.

The original version of this Article contained an error in the spelling of the author Xiaolong Jiang, which was incorrectly given as Xialong Jiang. This has now been corrected in both the PDF and HTML versions of the Article.

