# Supplementary material for: Layer 4 of mouse neocortex differs in cell types and circuit organization between sensory areas
Source: Nat Commun. 2019 Sep 13;10:4174. doi: 10.1038/s41467-019-12058-z (PMC6744474; doi:10.1038/s41467-019-12058-z)
Supplement: Supplementary file 1 — Supplementary Information [file 41467_2019_12058_MOESM1_ESM.pdf]

Layer 4 of mouse neocortex differs in cell types and circuit organization between sensory areas. Supplementary Information

Federico Scala, Dmitry Kobak, Shen Shan, Yves Bernaerts, Sophie Laturnus, Cathryn R. Cadwell, Leonard Hartmanis, Emmanouil Froudarakis, Jesus R. Castro, Zheng H. Tan, Stelios Papadopoulos, Saumil Patel, Rickard Sandberg, Philipp Berens, Xialong Jiang, Andreas S. Tolias

July 2019

Supplementary Note 1

The detailed description of interneuron cell types in V1 L4 is as follows.

Large basket cells (LBCs) in L4 were the most abundant interneuron type (37.6%, 88/234). Their morphology was very similar to BCs in L2/3 as described in our previous study (Jiang et al., 2015). Somata of these neurons were usually larger than those of other L4 neurons. Their dendrites projected vertically in a bitufted manner, without a complex horizontal structure. The most salient morphological feature of LBCs was a thick axon originating from the apical side of the soma. It typically projected towards L2/3 forming a series of major branches that extensively spread above the apical region of the soma, with few branches projecting horizontally and vertically downward to L5. All LBCs exhibited a fast-spiking (FS) firing pattern with narrow AP width and high maximal firing rate (Fig. 1b).

Martinotti cells (MCs) in L4 (20.1%, 47/234) were similar to those previously described both in the developing cortex and in the mature cortex in other layers (Wang et al., 2004; Bon-Jego and Yuste, 2007; Buchanan et al., 2012; Jiang et al., 2015). They had bitufted dendrites with vertically or obliquely oriented branches. All of them had an ascending axon that projected to L2/3 and L1, where it ramified horizontally and formed a dense axonal cluster of variable extension. A small subset of MCs (8.9%, 11 out of all 124 recovered MCs) showed a secondary axonal cluster within L4 (e.g. the last two MCs in Fig. 1a). Firing pattern and electrophysiological properties of MCs showed a strong correspondence to L2/3 and L5 MCs described in both adult (Jiang et al., 2015) and developing cortex (Wang et al., 2004). In particular, these neurons were distinguished from other interneurons by their large membrane time constant (tau, Fig. 1b).

Bipolar cells (BPCs) in L4 (12.4%, 29/234) had a small oval-shaped soma and bipolar dendrites projecting to L1 and L5. The ascending dendrites formed a tuft in L1, similar to the structure of apical dendrites of PYRs. However, their dendrites lacked dendritic spines. The axon emerged from one of the descending dendrites and projected predominantly to L5. All BPCs showed an irregular-firing pattern associated with a high input resistance and large AP amplitude (Fig. 1b).

Neurogliagorm cells (NGCs) in L4 (11.5%, 27/234) were very similar to NGCs in other layers with regard to their axonal and dendritic structures (Jiang et al., 2015). They were characterized by short and radially organized dendrites, and a very thin axon that highly ramified and formed a dense arborization around cell bodies. These neurons fired late-spiking action potentials with large AP width and high AP threshold (Fig. 1b).

Small basket cells (SBCs) in L4 (9.4%, 22/234) were similar to shrub cells that have been previously described in L5 of adult mouse (Jiang et al., 2015) and small basket cells in L2/3 and L4 of young rats (Wang et al., 2002). These neurons had non-polarized dendritic branches mostly residing in L4 and a thick axon often emerging from the apical side of the cell bodies and branching locally around their soma. All SBCs exhibited an FS firing pattern (Fig. 1b).

Double-bouquet cells (DBCs) in L4 (5.6%, 13/234) had large cell bodies and vertically-oriented bitufted dendrites, similar to LBCs. However, unlike LBCs, the thick axon often emerged from the bottom of the soma, projecting shortly towards L5 and forming several branches that projected upwards to L2/3 and downwards to L5 with variable distances. Notably, the axons of these cells extended also horizontally into L2/3 and L5, differing slightly from DBCs previously described in L2/3 (Jiang et al., 2015). All DBCs exhibited an FS firing pattern (Fig. 1b).

Horizontally elongated basket cells (HBCs) in L4 (3.4%, 8/234), with their horizontally extended axonal branches, were similar to the horizontally elongated cells previously reported in L5 (Muñoz et al., 2014; Jiang et al., 2015). In particular, the axon had a thick primary structure that often emerged from the apical side of the soma and bifurcated into secondary structures that spread horizontally mostly within L4. All HBCs exhibited an FS firing pattern (Fig. 1b).

Supplementary Figures

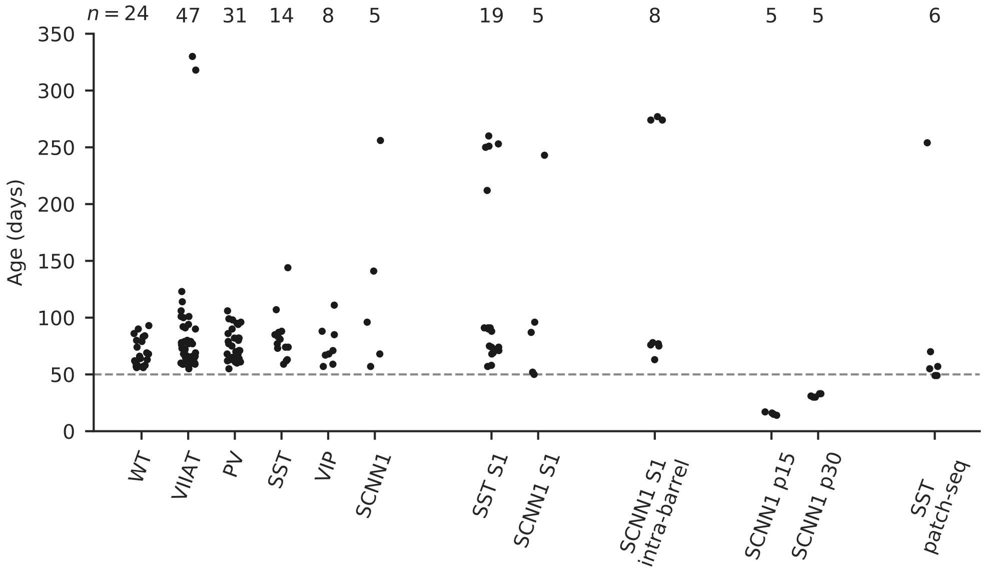

Supplementary Figure 1: Mice ages. WT stands for wild type, all other abbreviations correspond to Cre lines.

Morphology of labeled neurons for mouse Cre line

Cell type / Cre-line	PYR	LBC	MC	SBC	BPC	NGC	DBC	HBC	NMC	Ste
Viiat V1 (n=47)	**11/245 (4.5%)**	**88/245 (35.9%)**	**47/245 (19.2%)**	**22/245 (8.9%)**	**29/245 (11.8%)**	**27/245 (11.0%)**	**13/245 (5.3%)**	**8/245 (3.3%)**	/	/
PV V1 (n=31)	/	**126/163 (77.3%)**	/	**15/163 (9.2%)**	/	/	**20/163 (12.3%)**	**2/163 (1.2%)**	/	/
VIP V1 (n=8)	/	/	/	/	**28/28 (100%)**	/	/	/	/	/
SCNN1A V1 p15/20 (n=5)	**29/29 (100%)**	/	/	/	/	/	/	/	/	/
SCNN1A V1 p30/40 (n=5)	**48/48 (100%)**	/	/	/	/	/	/	/	/	/
SCNN1A V1 >p 60 (n=5)	**30/30 (100%)**	/	/	/	/	/	/	/	/	/
SOM V1 (n=14)	/	**5/61 (8.2%)**	**56/61 (91.8%)**	/	/	/	/	/	/	/
SOM S1 (n=19)	/	**1/79 (1.3%)**	**2/79 (2.5%)**	/	/	/	/	/	**76/79 (96.2%)**	/
SCNN1A S1 (n=5)	**11/43 (25.6%)**	/	/	/	/	/	/	/	/	**32/43 (74.4%)**

Supplementary Figure 2: Cre lines. Morphological types of labeled neurons in different mouse Cre lines.

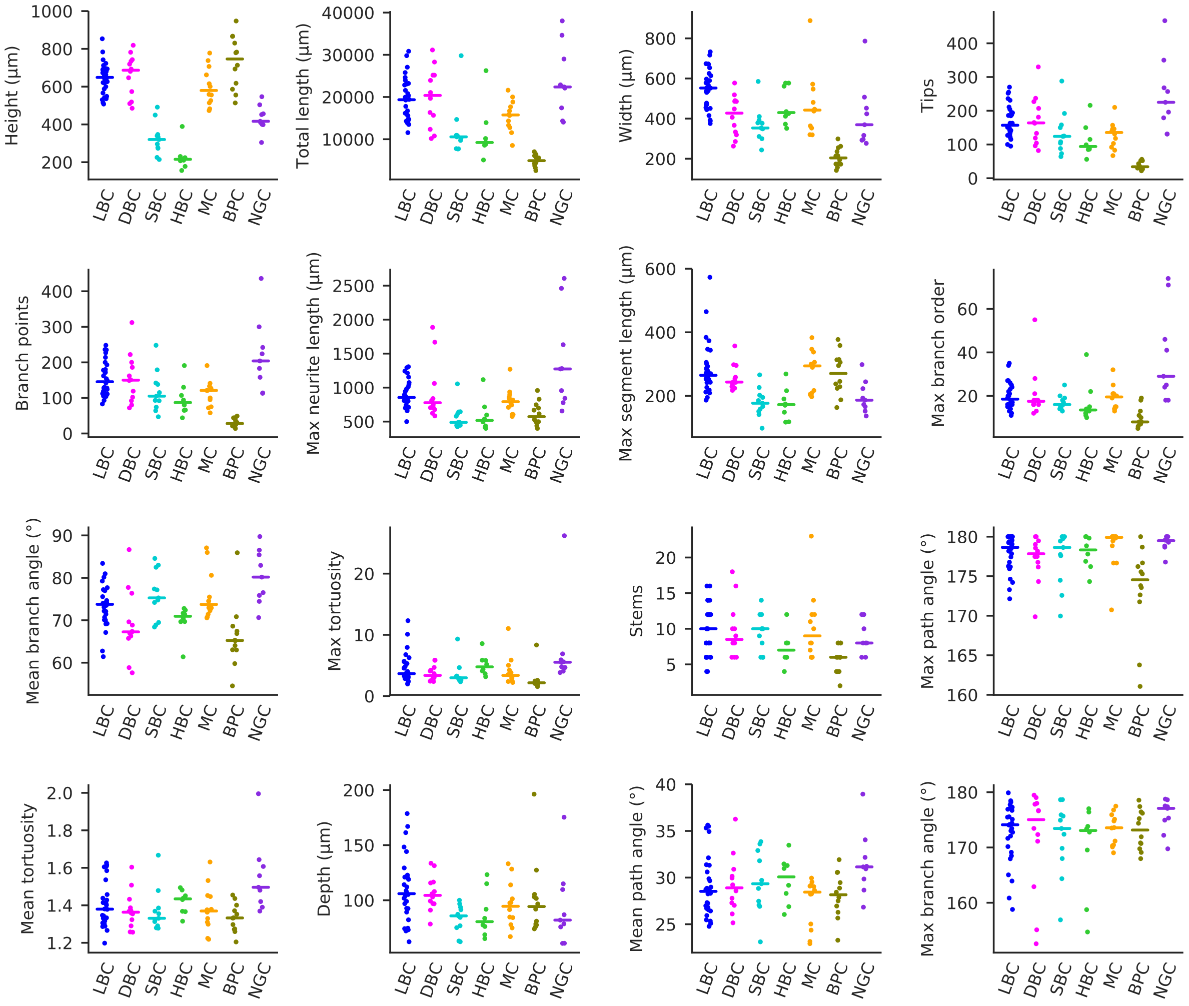

Supplementary Figure 3: Morphological features of neuronal cell types in V1 L4. Each panel shows one of the 16 selected morphological summary statistics for $n = 92$ neurons: height (extent along the cortical depth), total length of neurites, width (extent along the x-axis), number of tips, number of branch points, length of the longest neurite from tip to soma, length of the longest segment, maximum branch order, mean branch angle between two branches, maximum tortuosity, maximum path angle, number of stems extending from the soma, mean tortuosity, depth (extent along the y-axis), mean path angle and maximum branch angle. Features are sorted by how strongly they varied between cell types (from strongest to weakest), as quantified by the Kruskal-Wallis test statistic. Horizontal lines show medians in each cell type.

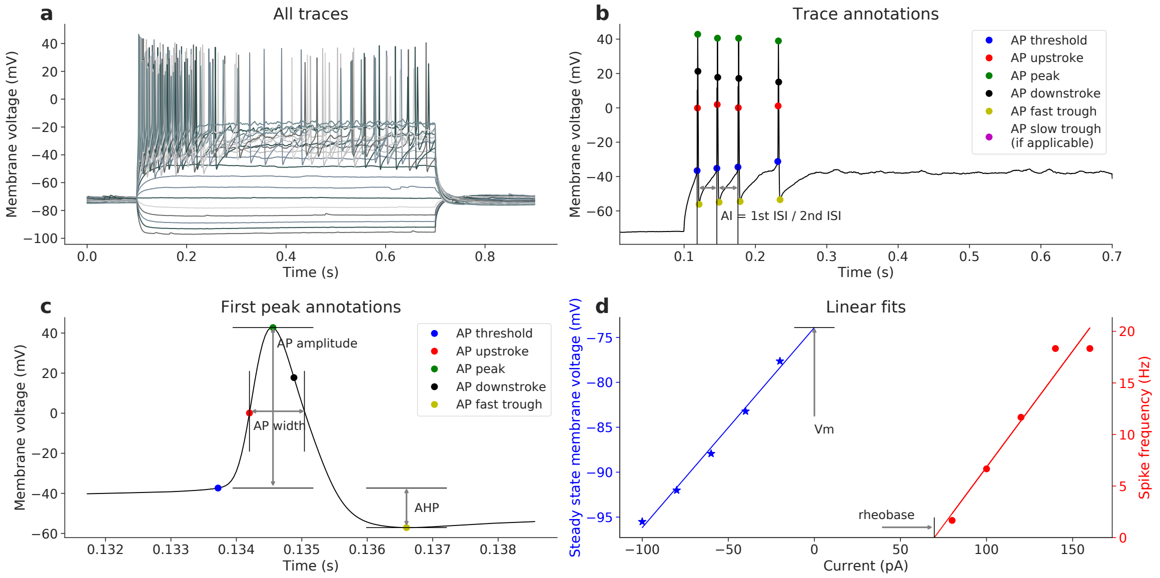

Supplementary Figure 4: Schematic of the electrophysiological features. All panels show data from the same exemplary Martinotti cell. **(a)** Membrane potential responses to the consecutive step current injections. The maximum number of action potentials (APs, or spikes) elicited in 600 ms was 11. Hyperpolarizing currents are used to compute sag ratio (1.3) and membrane time constant tau (23.2 ms). **(b)** Zoom-in to one particular trace in (a) showing trace annotations and AI (1.13). **(c)** Zoom-in to the first AP elicited in this neuron. This action potential is used to compute AP threshold (−40.1 mV), AP amplitude (71.3 mV), AP width (0.72 ms), AHP (−14.1 mV), ADP (6.6 mV), and latency of the first spike (78.7 ms). **(d)** Blue regression line gives an estimate of resting membrane potential (−58.9 mV) and input resistance (235.8 MΩ). Red regression line gives a rheobase estimate (40 pA).

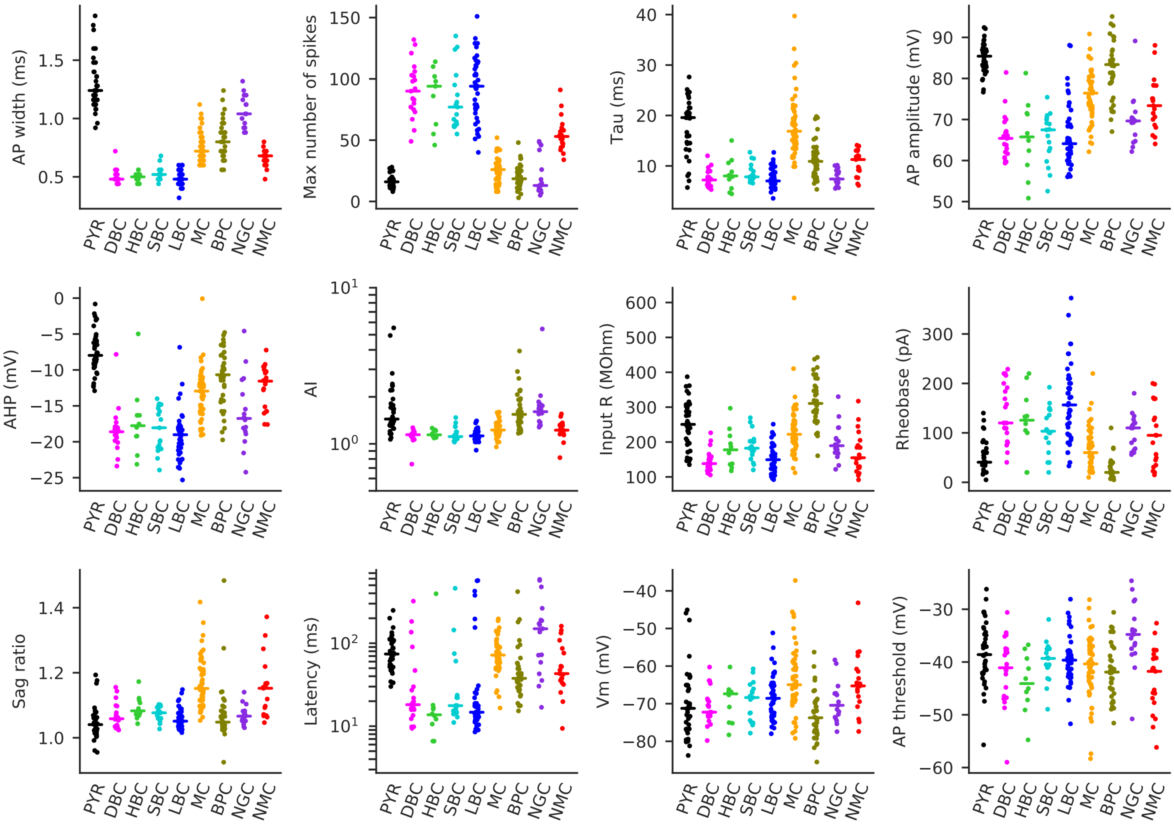

Supplementary Figure 5: Electrophysiological features of neuronal cell types. All cell types apart from NMC (red) are from V1 L4. NMC is from S1 L4. Each panel shows one of the 13 automatically extracted electrophysiological features for n=254 neurons: action potential (AP) width, maximum number of APs emitted during 600 ms of stimulation, membrane time constant tau, AP amplitude, afterhyperpolarization (AHP) depth, input resistance, adaptation index, rheobase, sag ratio, latency of the first AP, membrane potential, and AP threshold. Features are sorted by how strongly they varied between cell types (from the most strongly to the least strongly), as quantified by the Kruskal-Wallis test statistic. Horizontal lines show medians in each cell type. Afterdepolarization (ADP) height is not shown because its median was 0 for all cell types. See Supplementary Figure 4 for explanations of the features.

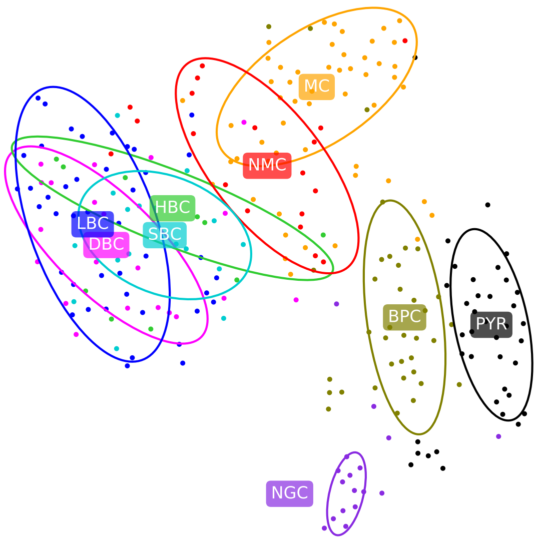

Supplementary Figure 6: t-SNE in the electrophysiological space. This figure is analogous to Fig. 2b, but includes $n = 19$ NMCs from S1 in addition to the $n = 235$ cells from V1.

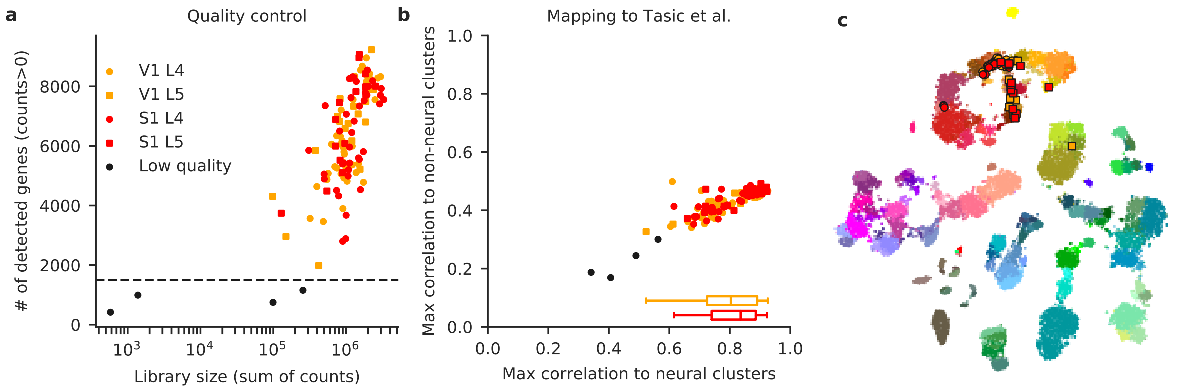

Supplementary Figure 7: Quality control of Patch-seq data. (a) Distribution of library sizes (total sum of gene counts) and numbers of detected genes (number of positive counts) for each sequenced cell ($n = 118$). Four cells with less than 1500 genes detected were excluded. **(b)** For each cell, we found its maximal correlation to the cluster means of the Tasic et al. (2018) dataset across all neural clusters (x-axis) and across all non-neural clusters (y-axis). Boxplots show distribution of maximal correlation for V1 and S1 cells (excluding the low quality cells). Correlations were not lower for S1 cells, despite the fact that the Tasic et al. dataset only contained data from V1 and ALM. **(c)** All $n = 114$ remaining cells were positioned on the t-SNE map of the Tasic et al. dataset. Three cells mapped to Pvalb clusters and one cell mapped to excitatory clusters.

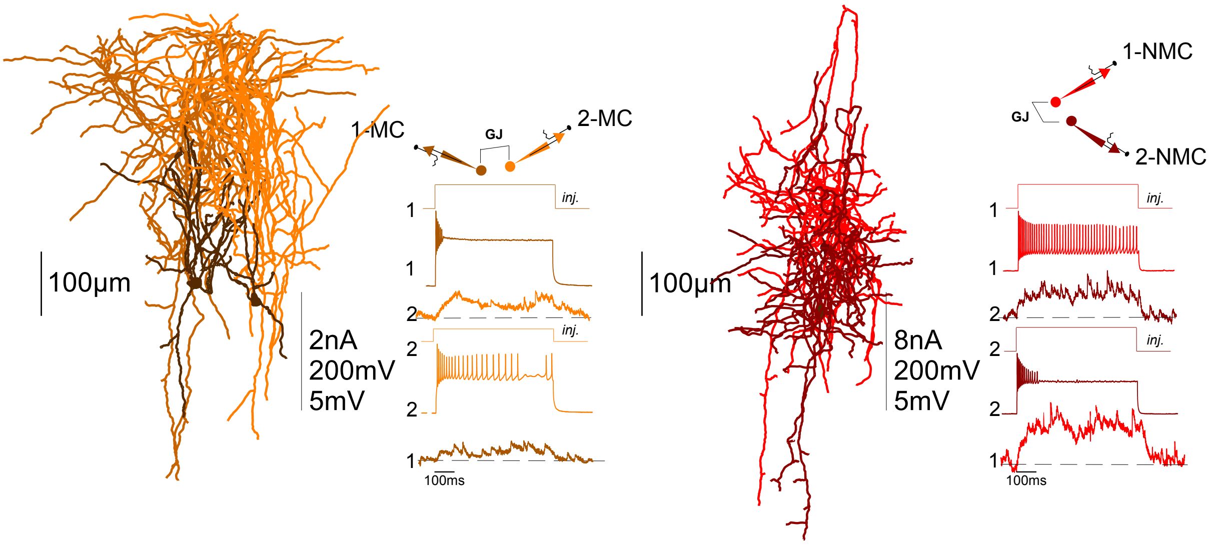

Supplementary Figure 8: Gap junctions between MCs and NMCs. Simultaneous recordings of two L4 MCs in V1 (left) and simultaneous recordings of two L4 NMCs in S1 (right). Depolarizing current injections into either MC (left) or NMC (right) were transmitted to the other cell, confirming electrical coupling (the traces in the right side). The percentage of gap junctions was 23.5% in V1 (8/34) and 30.7% in S1 (8/26).

uI/EPSP (mV) in V1

POST \ PRE	PYR	LBC	MC
**PYR**	***0.75±0.13 mV*** *PPR=75.6±5.1%*	***0.68±0.07 mV***	***0.50±0.07 mV***
**LBC**	***1.17±0.23 mV*** *PPR=85.8±6.5%*	***0.77±0.05 mV***	***0.59±0.09 mV***
**MC**	/	***0.53±0.15 mV***	/

uI/EPSP (mV) in V1 among age

	PYR-PYR
p15/20	***0.69±0.15 mV*** *PPR=78.9±8.7%*
p30/40	***0.58±0.19 mV*** *PPR=73.2±6.6%*
adult	***0.75±0.13 mV*** *PPR=75.6±5.1%*

uI/EPSP (mV) in S1

POST \ PRE	EXC	NMC
**EXC**	***0.99±0.34 mV*** *PPR=87.2±4.0%*	*0.38±0.12 mV*
**NMC**	***0.53±0.16 mV*** *PPR=139.±17.0%*	*0.19±0.05 mV*

uI/EPSP (mV) in S1 intra-barrel

	EXC
**EXC**	***0.32±0.09 mV*** *PPR=69.1±4.6%*

Supplementary Figure 9: Amplitudes of unitary inhibitory and excitatory postsynaptic potentials (uIPSPs/uEPSPs) and paired-pulse ratios (PPR).

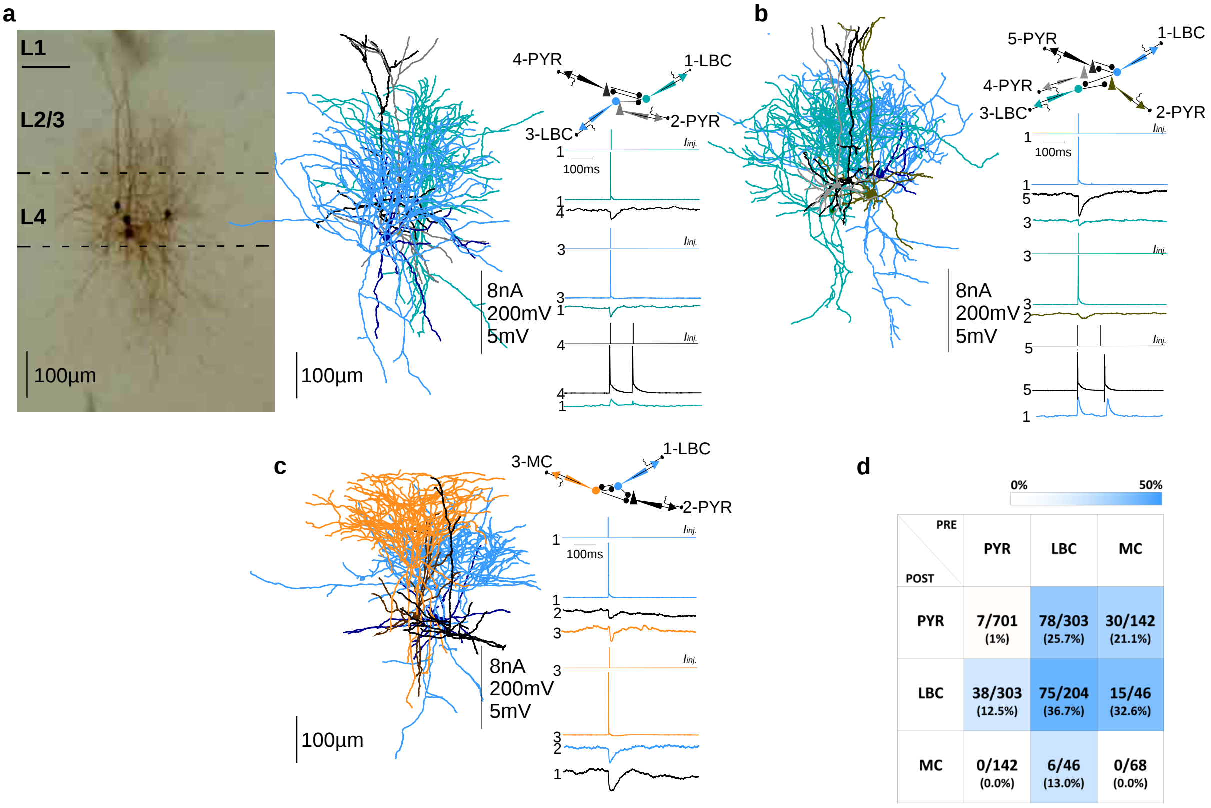

Supplementary Figure 10: Connectivity between L4 PYRs, LBCs, and MCs in V1. **(a)** On the left: Example of morphological recovery of four simultaneously-recorded neurons. Recorded neurons were close to each other (generally less than 150 µm). On the right: connection diagram of the same neurons, including two LBCs and two PYRs, and their reconstructed morphology. Vertical scale bar indicates: amplitudes of injected current in nA, amplitude of APs in mV and amplitude of uEPSP or uIPSP in mV. **(b)** Connections between five simultaneously-recorded neurons including three PYRs and two LBCs. **(c)** Connections between three simultaneously-recorded neurons including one PYR, one LBC and one MC. **(d)** Color coded connectivity matrix showing the connection probabilities between PYRs, LBCs and MCs computed as a fraction of all potential connections tested. Unitary inhibitory and excitatory postsynaptic potentials and paired-pulse ratios are reported in Supplementary Figure 9.

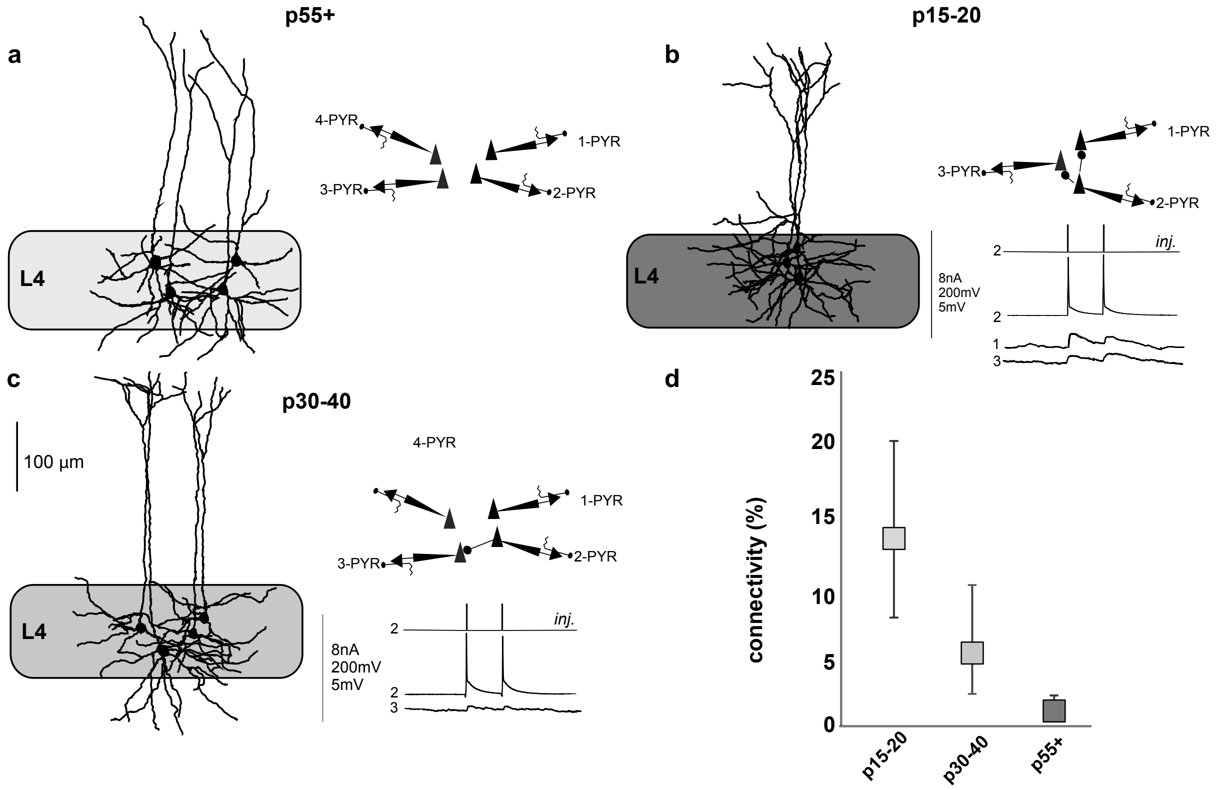

Supplementary Figure 11: Connectivity between L4 PYRs in V1 at different ages. (a–c) Examples of simultaneously-recorded L4 neurons in P55+ (a), P15–20 (b), and P30–40 (c) mice. **(d)** Connection probability between PYRs at different ages: 13.2% in P15–20 (15/114), 5.1% in P30–40 (8/156), and 1.0% in P55+ with median age P71 (7/701). Error bars are 95% Clopper-Pearson confidence intervals.

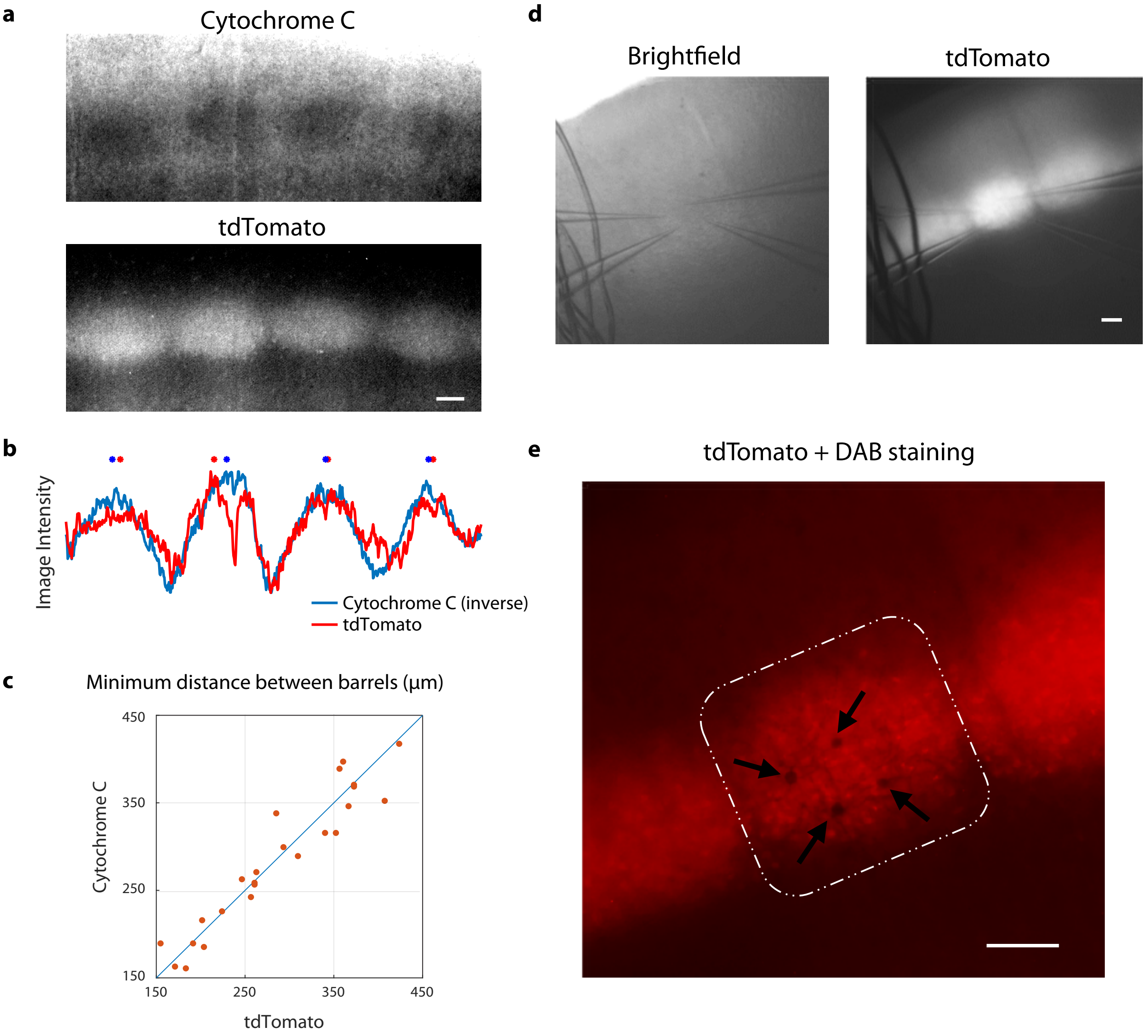

Supplementary Figure 12: Identification of L4 barrels in S1 for intra-barrel recordings. **(a)** One exemplary slice showing barrels in L4 of somatosensory cortex identified by either cytochrome C staining (above) or by tdTomato fluorescence (below) in Scnn1a-Cre mouse. **(b)** Average intensity for the Cytochrome C and tdTomato images shown in (a). Note that the traces shown here are normalized (between 0 and 1) and high-pass filtered but not smoothed. The signal of the cytochrome C was inverted for easier comparison to the fluorescence trace. The barrel centers, indicated with asterisks, were detected as the peaks of intensity of the smoothed traces. **(c)** Distances between adjacent barrels detected with either Cytochrome C or tdTomato. Summary across $n = 7$ slices. **(d)** An example of intra-barrel quadruple recording. Left: under brightfield. Right: tdTomato signal. **(e)** Diaminobenzidine (DAB) staining confirmed the intra-barrel localization of the recorded neurons. All scale bars represent 100 µm.

Supplementary References

Le Bon-Jego and Rafael Yuste. Persistently active, pacemaker-like neurons in neocortex. *Frontiers in Neuroscience*, 1:9, 2007.

Katherine A Buchanan, Arne V Blackman, Alexandre W Moreau, Dale Elgar, Rui P Costa, Txomin Lalanne, Adam A Tudor Jones, Julia Oyrer, and P Jesper Sjöström. Target-specific expression of presynaptic NMDA receptors in neocortical microcircuits. *Neuron*, 75(3):451–466, 2012.

Xiaolong Jiang, Shan Shen, Cathryn R Cadwell, Philipp Berens, Fabian Sinz, Alexander S Ecker, Saumil Patel, and Andreas S Tolias. Principles of connectivity among morphologically defined cell types in adult neocortex. *Science*, 350(6264):aac9462, 2015.

William Muñoz, Robin Tremblay, and Bernardo Rudy. Channelrhodopsin-assisted patching: in vivo recording of genetically and morphologically identified neurons throughout the brain. *Cell Reports*, 9 (6):2304–2316, 2014.

Bosiljka Tasic, Zizhen Yao, Lucas T Graybuck, Kimberly A Smith, Thuc Nghi Nguyen, Darren Bertagnolli, Jeff Goldy, Emma Garren, Michael N Economo, Sarada Viswanathan, et al. Shared and distinct transcriptomic cell types across neocortical areas. *Nature*, 563(7729):72, 2018.

Yun Wang, Anirudh Gupta, Maria Toledo-Rodriguez, Cai Zhi Wu, and Henry Markram. Anatomical, physiological, molecular and circuit properties of nest basket cells in the developing somatosensory cortex. *Cerebral Cortex*, 12(4):395–410, 2002.

Yun Wang, Maria Toledo-Rodriguez, Anirudh Gupta, Caizhi Wu, Gilad Silberberg, Junyi Luo, and Henry Markram. Anatomical, physiological and molecular properties of Martinotti cells in the somatosensory cortex of the juvenile rat. *The Journal of Physiology*, 561(1):65–90, 2004.
